# The genus *Arctorthezia* Cockerell (Hemiptera, Ortheziidae) with the description of a new species

**DOI:** 10.3897/zookeys.472.8928

**Published:** 2015-01-19

**Authors:** Éva Szita, Mehmet Bora Kaydan, Zsuzsanna Konczné Benedicty, Hirotaka Tanaka, Kinga Fetykó, Ferenc Kozár

**Affiliations:** 1 Plant Protection Institute, Centre for Agricultural Research, Hungarian Academy of Sciences H-1022, Herman Ottó út 15, Budapest, Hungary; 2 Çukurova Üniversity, Imamoglu Vocational School, Adana, Turkey; 3 Tottori Prefectural Museum, Higashi-machi 2-124, Tottori-shi, Tottori-pref., 680-0011 Japan

**Keywords:** Ensign scale, archaeococcoids, taxonomy, distribution, Palaearctic Region, Switzerland

## Abstract

This paper describes a new species of *Arctorthezia* Cockerell (Hemiptera: Coccoidea: Ortheziidae) from the Palaearctic region. The specimens were extracted from forest litter in the collections of Muséum d’histoire Naturelle de Genève, Switzerland, using Berlese funnels. Three further species, *Arctorthezia cataphracta* (Olafsen), *Arctorthezia occidentalis* (Douglas) and *Arctorthezia pseudoccidentalis* Morrison, are redescribed and re-illustrated. The genus *Arctorthezia* now contains five species. An identification key, diagnostic illustrations, photographs of unmounted females and new locality records of the *Arctorthezia* species currently known are provided.

## Introduction

Ensign scale insects (Hemiptera: Coccoidea: Ortheziidae) are considered to be one of the most ancient families of the Coccoidea (Koteja 1996, Vea and Grimaldi 2012) and are thought to be either ‘ancestral’ to all scale insects or a ‘primitive’, isolated branch of the grade of families, the archaeococcoids (Vea and Grimaldi 2012). Ensign scales are readily distinguished by (i) possessing distinctive, well-developed legs and antennae, (ii) having much of the body cloaked in bunches of white wax secretions (Vea and Grimaldi 2012). There are approximately 212 described species of Ortheziidae to date, classified in 21 genera (including four extinct genera) (Vea and Grimaldi 2012). Only a few species of ortheziids are serious pests; these include *Insignorthezia insignis* (Browne), the greenhouse ensign scale, and an invasive pest in Afro-tropical region, *Praelongorthezia praelonga* (Douglas), the citrus orthezia (Kondo et al. 2013). In Ortheziidae, two main groups of host plant specialization can be observed; the first group is composed of species that occur in leaf litter (feeding on roots and fungal mycelia), and on mosses and lichens; the second group feeds on vascular plants, including grasses, herbaceous and woody plants (Koteja 1996, Kozár 2004, Vea and Grimaldi 2012).

The subfamily Ortheziinae is characterized by having the tibia and tarsus well separated, and 7- or 8-segmented antennae (Kozár 2004). Two tribes can be recognized in the subfamily, namely Arctortheziini Kozár and Ortheziini Amyot & Serville. The Arctortheziini includes species living in moist habitats – in litter, moss and feeding on the roots of different plants; whereas the Ortheziini includes species feeding on different kinds of plants (grasses, herbaceous, woody plants), mostly living in dry habitats. The monotypic tribe Arctortheziini includes only one genus, *Arctorthezia* Cockerell, which contains four living species: *Arctorthezia cataphracta* (Olafsen), *Arctorthezia occidentalis* (Douglas), *Arctorthezia pseudoccidentalis* Morrison and *Arctorthezia vardziae* Hadzibejli (Richard 1998) and one extinct species (Koteja and Zak-Ogaza 1988). *Arctorthezia* is characterized by (i) 9 pairs of dorsal wax plates in each marginal row, 8 pairs in the submedian band and 3 triangular or shield-shaped plates in the center of the thorax; (ii) 7- or 8-segmented antennae; (iii) 7 or 8 pairs of abdominal spiracles, and (iv) 3 or 4 spine rows within the ovisac band.

In the present paper, one new *Arctorthezia* species is described from the Palaearctic region (Switzerland); three species, namely *Arctorthezia cataphracta* (Olafsen), *Arctorthezia occidentalis* (Douglas), *Arctorthezia pseudoccidentalis* Morrison, are redescribed and re-illustrated; an identification key and new additional locality records for the currently known *Arctorthezia* species are provided; and macromorphological characters are illustrated and discussed.

## Material and methods

The specimens examined in this study were mostly from the scale insect collections of the Plant Protection Institute, Centre for Agricultural Research, Hungarian Academy of Sciences (PPI) and the Muséum d’histoire Naturelle de Genève (MHNG). Important material was processed from the Soil Zoology Collection of the Hungarian Natural History Museum, Budapest (HNMH), and part of the material was loaned from the United States National Entomological Collection, Washington (USNM). The material examined from Japan belongs to the collection of Tottori Prefectural Museum, Japan (TRPM).

Specimens were prepared for light microscopy using the slide-mounting method described by Kosztarab and Kozár (1988). The morphological terminology used follows Kozár (2004).

The holotype of the new species is deposited in the Muséum d’histoire Naturelle de Genève collection (MHNG), and the paratypes are shared between MHNG and PPI.

The digital images of unmounted females were made with a Canon Eos400D camera and an MBC-10 stereomicroscope, and focus-stacking was processed by CombineZP software (Hadley 2010).

Measurements and counts were taken from all available material, and the values are given as a range for each character.

## Result and discussion

### Genus Arctorthezia

Taxon classificationAnimaliaHemipteraOrtheziidae

Cockerell, 1902

Orthezia (Arctorthezia) Cockerell 1902: 114. Type species: *Orthezia occidentalis* Douglas, 1891.
Arctorthezia , Morrison 1925: 143 (as subgenus). Change of status.
Arctorthezia , Morrison 1952: 53 (as genus). Change of status.

#### Type species.

*Orthezia occidentalis* Douglas, 1891: 245. Subsequently designated by Cockerell 1902: 259.

#### Comments.

Cockerell (1902) described *Arctorthezia* as a “section” of *Orthezia*. According to the International Code of Zoological Nomenclature, Article 10 (e) a “section” is deemed to be a subgeneric name when proposed for a species-group division of a genus. Thus, Cockerell is considered to be the author of *Arctorthezia* (Kozár 2004).

#### Description.

*Unmounted female.* Live adult female with 9 pairs dorsal wax plates in each marginal row, 8 pairs in each submedian band, and 3 triangular or shield-shaped plates in centre of thorax. Ovisac parallel sided, short, about half length of body (Fig. 5).

*Mounted female.* Antenna 7 or 8 segmented, covered with strong spines; apical seta a blunted strong spine. Claw without denticle. Abdominal spiracles numbering 7 or 8 pairs, situated on margin of dorsum. Three triangular wax plates present on mid-dorsum. Three or four rows of spines present within ovisac band.

#### Host plant.

Found under mosses and stones on the roots of different plants, also reported from ant nests.

#### Distribution.

The known species are distributed only in the Holarctic Region.

#### Key to adult females of species of *Arctorthezia*

Unfortunately the type material of *Arctorthezia vardziae* was not available. For this reason we could not include this species in the key. In the original description, Hadzibejli (1963) only provided generic characters that refer to the genus description, so further studies are needed to clarify the species concept of *Arctorthezia vardziae*.

**Table:** 

1	Triangular wax plate on mid-dorsum longer than wide; triangular wax plate between mid coxae on venter longer than wide, like those on dorsum; ovisac band containing 3 wide wax plate bands	***Arctorthezia cataphracta***
–	Triangular wax plate on mid-dorsum various widths but never the longer than wide; triangular wax plate between mid-coxae on venter at least twice as wide as long; ovisac band containing 4 wide wax plate bands	**2**
2	On the dorsum, with 9 circular discoidal pore plates present between marginal and submedian setal plates (Fig. 4)	***Arctorthezia pseudoccidentalis***
–	On the dorsum, circular discoidal pore plates absent from between marginal and submedian setal plates	**3**
3	Dorsal triangle-shaped mid-thoracic setal plates more than two times wider than long (Fig. 6e); distal to vulva, proportion of simple pores to quadrilocular pores ca. 1:6	***Arctorthezia occidentalis***
–	Dorsal triangle-shaped mid-thoracic setal plates hardly wider than long (Fig. 6a); distal to vulva, proportion of simple pores to quadrilocular pores ca. 1:15	***Arctorthezia helvetica* sp. n.**

### Arctorthezia helvetica

Taxon classificationAnimaliaHemipteraOrtheziidae

Kozár & Szita sp. n.

http://zoobank.org/AFE6A314-D608-4728-B3CD-BB3E2BFDF8EE

Figs 1 , 5a , 6a–b

#### Material.

Type material: Holotype female Switzerland, Valais, s/Venayaz, 700 m a.s.l., 7.x.1980, leg. C. Besuchet [PPI 8864, MHNG without code]. Paratypes female: 6 adult females with the same data as holotype, on separate slides.

Other material examined: 3 adult females on separate slides, Switzerland, Valais, Dorenaz, oak, 28.iii.1964, leg. C. Besuchet [PPI 8862, MHNG without code].

#### Description.

Live adult female: dorsum with 9 pairs dorsal wax plates in each marginal row, 8 pairs in each submedian band, and 3 triangular or shield-shaped plates in middle of thorax. Ovisac short, about half length of body (Fig. 5a).

**Figure 1.**
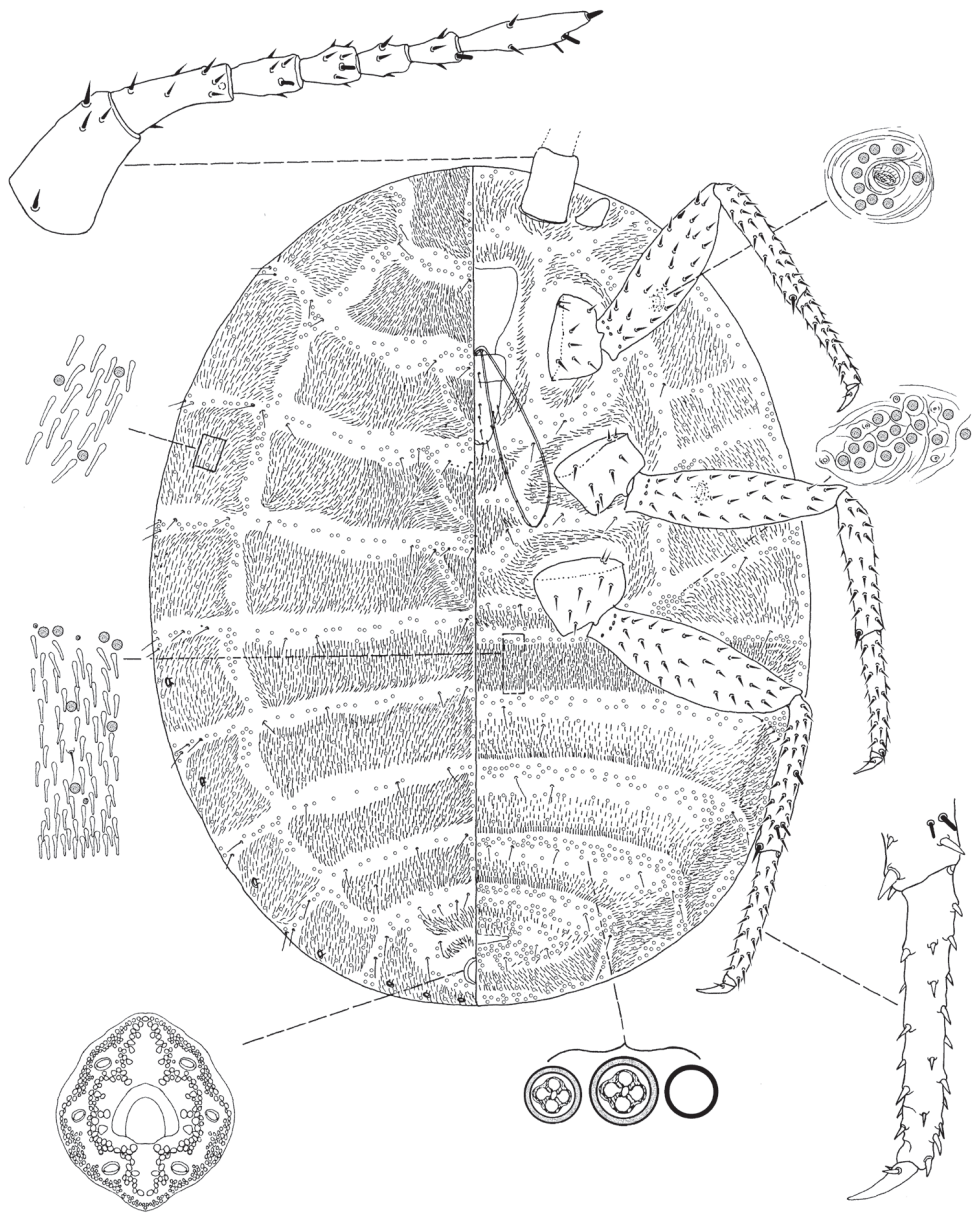
*Arctorthezia helvetica* sp. n., mounted adult female. Illustration by Konczné Benedicty & Kaydan.

*Slide-mounted adult female* (Figs 1, 6a–b). Body elongate oval, 2.124‒2.719 mm long, 1.425‒1.942 mm wide. Antenna 7 segmented. Measurements of antennal segments: 1^st^ 180‒230, 2^nd^ 183‒209, 3^rd^ 96‒117, 4^th^ 84‒107, 5^th^ 76‒92, 6^th^ 76‒96 and apical 204‒220 mm long, apical spine of antenna 22‒24 mm long, subapical seta absent; fleshy sensory seta near apical seta 22‒24 mm long; all segments of antenna covered with very robust, spine-like setae, the longest 17 mm long; first antennal segment with 4 spines on each side of segment. Eyes sub-conical, well separated from base of first antennal segment.

*Venter*: Labium 260‒285 mm long, apparently one segmented. Stylet loop usually longer than labium. Legs well developed; leg measurements: front coxa 155‒189 mm, middle 155‒189 mm, hind 188‒204 mm; front trochanter+femur 546‒577 mm long, middle 548‒572 mm, hind 597‒650 mm; front tibia+tarsus 700‒722 mm long, middle 679‒740 mm, hind 804‒866 mm; claw 74‒89 mm long; hind claw digitules each 15‒21 mm. Legs with rows of robust spine-like setae, tibia with fleshy sensory seta 24 mm long, trochanter with 4 placoid sensilla on each surface. Wax plates present on ventral areas of head and thorax, with wide marginal plate laterad of each thoracic spiracle; two wax plates present between mid-coxae, both triangular, almost same size (Fig. 6a). Thoracic spiracle with wide band of disc pores inside atrium. Setae few, scattered in medial areas of thorax; several setae near anterior edge of ovisac band and associated with simple pores. Ovisac band wide, parallel side in the middle, with 4 spine rows within ovisac band. Multilocular pores with 4 loculi around perimeter, each loculus 4‒5 mm in diameter and 1 loculus in central hub, 3 mm in diameter; pores present in 3 complete bands near posterior edges of each spine band, and scattered around vulva.

*Dorsum*: Wax plates covering entire dorsum; three triangular wax plates on mid-dorsum, anteriormost 185‒190 mm wide, 135‒150 mm long; middle 250‒270 mm wide, 150‒160 mm long; posteriormost 260‒280 mm wide, 170‒180 mm long (Fig. 6b). Spines at margin of each wax plate each 22‒24 mm long, apically capitate. A few setae present in marginal clusters near posterior edges of marginal wax plates; 3‒6 setae present laterad of thoracic spiracle, longest seta 24 mm long; also present in very small numbers on other wax plates and in medial bare area. Multilocular pores generally with 4 loculi around perimeter and one loculus in central hub, sparsely present in wax plates. Abdominal spiracles numbering 8 pairs on the margin, last 3 pairs situated in posterior apical spine clusters. Anal ring 108‒120 mm long, 108‒120 wide, bearing 6 blunt anal ring setae, each 156‒192 mm long.

#### Etymology.

he species was named after the ancient name of its *locus typicus*, Switzerland (Helvetia).

#### Distribution.

Switzerland.

#### Ecology.

*Host plant*: unknown, found in leaf litter and soil samples.

#### Diagnosis.

*Arctorthezia helvetica* sp. n. can be recognized by the following combination of characters: (i) 7-segmented antennae, (ii) dorsal triangle-shaped mid-thoracic setal plates hardly wider than long (iii) the proportion of simple pores and quadrilocular pores distal to vulva ca. 1:15, (iv) diamond-shape setal plate between mid-coxae on venter.

#### Comments.

*Arctorthezia helvetica* sp. n. is closest to *Arctorthezia occidentalis* in having 4 spine rows within the ovisac band and lacking circular pore clusters on the dorsum, but differs from *Arctorthezia occidentalis* as follows (characters of *Arctorthezia occidentalis* in brackets): (i) adult female body length less than 3 mm (adult female body length at least 3.5 mm); (ii) the fourth spine row within ovisac band weak (the fourth spine row within ovisac band strong); (iii) the proportion of simple pores to quadrilocular pores distal to vulva ca. 1:15 (the proportion of simple pores to quadrilocular pores distal to vulva ca. 1:6); (iv) dorsal triangle-shaped mid-thoracic setal plates hardly wider than long (dorsal triangle-shaped mid-thoracic setal plates more than two times wider than long).

### Arctorthezia cataphracta

Taxon classificationAnimaliaHemipteraOrtheziidae

(Olafsen, 1772)

**Figure 2.**
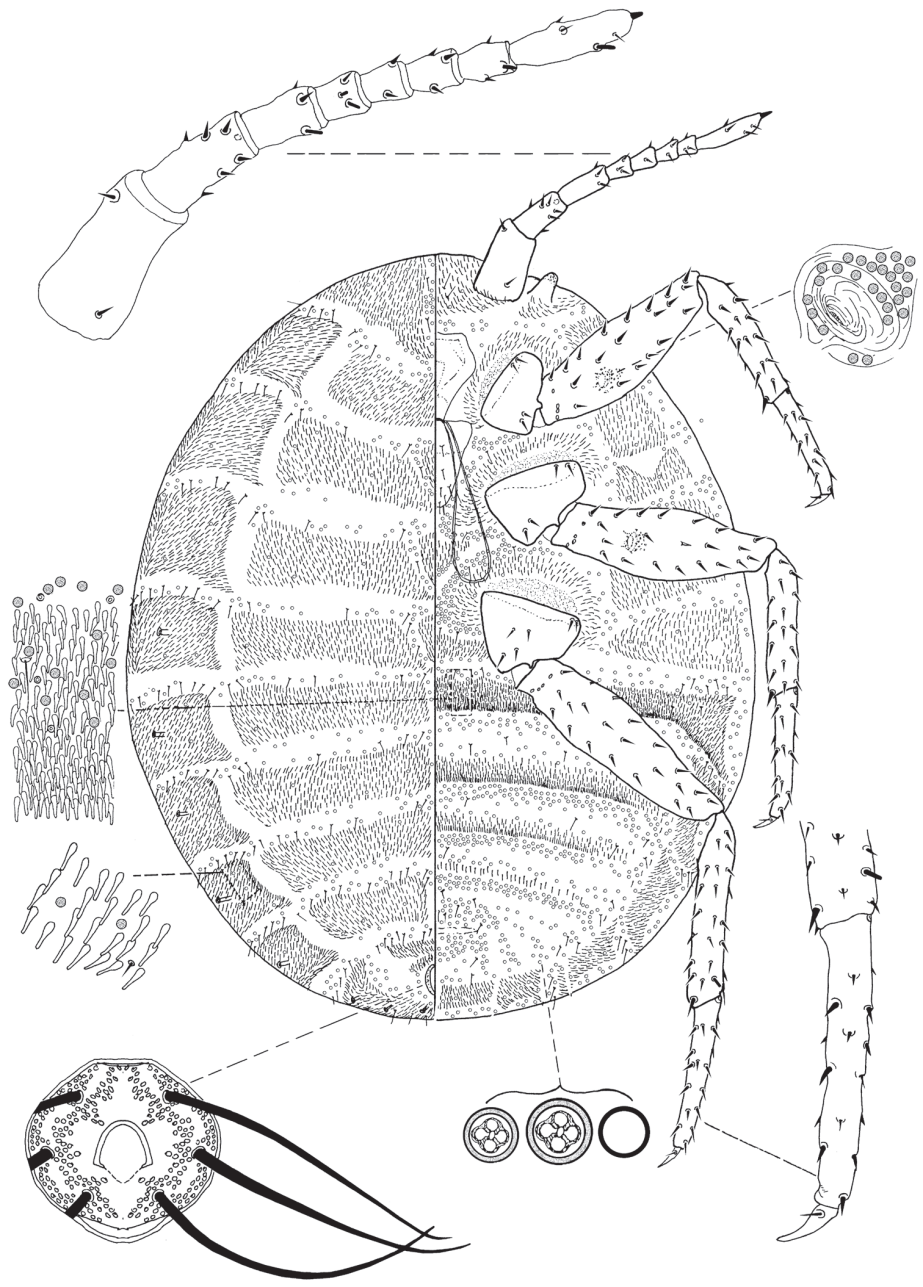
*Arctorthezia cataphracta* (Olafsen, 1772), mounted adult female. Illustration by Konczné Benedicty & Kaydan.

Figs 2 , 5b–c , 6c–d

#### Material examined.

**Austria:** 1 female, Kesselspitze Mt., 3.vii.1999, leg. K. Thaler [PPI 6545]. **Greenland:** 2 females on 1 slide, s-o, Nanortalik, dwarf willow, 26.vii.1979, leg. G. Primatesta [PPI 8933, MHNG nr.7]; 1 female at Boston, *Sedum* sp., 6.vi.1945, leg. Hodson [PPI 8967, USNM 45 1781]. **Italy:** 2 females on 2 slides, Piedmont, Monte Autoroto, 1700 m a.s.l., 16.vi.1982, leg. A. Focarile [PPI 9854, MHNG 13]. **Japan:** 1 female, Tochigi, Nikkô, Kawamata, Nikkô-zawa-onsen, 1500 m a.s.l., *Dryopteris crassirhizoma*, 5.vi.2013., leg. S. Maehara. (collected by beating) [TRPM]; 1 female, same locality, *Dryopteris crassirhizoma*, 2.vii.2013, leg. S. Maehara. (collected by beating) [TRPM]. **Mongolia:** 1 female, Bogdo, 3.vi.1967, leg. Z. Kaszab [HNHM As 77]. **Switzerland:** 2 females on 2 slides, Valais, Gornergat, 3050 m a.s.l., under stones, 12.ix.1982, leg. C. Besuchet [PPI 8857, MHNG without code]; 3 females on 3 slides, Valais, Fluhalp, near Leuerbad, 2000 m a.s.l., mosses and dead leaves, 14.viii.1980, leg. C. Besuchet [PPI 8858, MHNG without code]; 2 females on 2 slides, Obwald, Melchsee, 1800 m a.s.l., mosses, 2.x.1987, leg. I. Löbl [PPI 8861, MHNG without code]; 4 females on 4 slides, Valais, Tursten, s/Zermatt, 2200 m a.s.l., 14.vii.1966, leg. A. Comellini [PPI 8863]; 5 females on 5 slides, Valais, Saas-Almagell, 1650 m a.s.l., wet mosses, 5.vii.1997, leg. C. Besuchet [PPI 8866, MHNG without code]; 2 females on 2 slides, Valais, Grand St. Bernard, 2150 m a.s.l., mosses at foot of rocks, 10.ix.1996, leg. C. Besuchet [PPI 8873, MHNG without code]; 1 female, at N.Y., on lichens around *Rhododendron*, 12.vii.1938, leg. Harley [PPI 8968, USNM 98-2244, NY77615]; 1 female, Valais, Torrenthorn, near Lukerbad, 2500-2600 m a.s.l., alpine meadows, 12.viii.1980, leg. C. Besuchet [PPI 8870, MHNG without code]; 1 female, Studen, moss, 20.ix.1992, leg. F. Kozár [PPI 4071]; 1 female, Romoos, Weise, 23.viii.1994, leg. Rézbányai [PPI 6144, HNHM]; 2 females on 1 slide, Romoos, Weise, 28.iv.1994, leg. Rézbányai [PPI 6143]. **Turkey:** 1 female, Ilgardagi Gecidi, 2350 m a.s.l., 13.vi.1986, leg. Löbl, Besuchet, Burckhardt [PPI 8881, MHNG without code]; 2 females on 2 slides, Kars, Ilgandas, Gecidi, on herbs and flowers, 13.vi.1986, leg. Löebl & Bernhardt [MHNG 10699/1-2 Ar.tube II]. **UK:** 2 females on 1 slide, Scotland, N. Berwich, E. Lothian, under moss,. ix.1926, leg. E.E. Green [PPI 8965, USNM without code]; 1 female, Scotland, Braemar, at base of grass, 8.viii.1979, leg. J.N. Cox [USNM 5/79].

#### Synonymy.

*Pediculus cataphractus* Olafsen, *Coccus cataphracta* (Shaw), *Dorthezia cataphracta* (Shaw), *Orthezia cataphracta* (Shaw), *Dorthesia chiton* Zetterstedt, *Orthezia signoreti* White, *Coccus uva* (Modeer), *Orthezia uva* (Modeer), Orthezia (Arctorthezia) cataphracta, Cockerell, *Coccus cataphractus* Lindinger (Ben-Dov et al. 2014, Kozar 2004).

#### Description.

Live adult female: dorsum with 9 pairs dorsal wax plates in each marginal row, 8 pairs in each submedian band, and 3 small triangular or shield-shaped ones in middle of thorax. Ovisac parallel sided, short, about half length of body (Fig. 5b–c).

*Slide-mounted adult female* (Figs 2, 6c–d). Body elongate oval, 2.0‒2.9 mm long, 1.5‒2.7 mm wide. Antenna 8 - (rarely 6 or 7) segmented. Measurements of antennal segments: 1^st^ 189–273, 2^nd^ 144–210, 3^rd^ 102–145, 4^th^ 64–110, 5^th^ 61–93, 6^th^ 67–90, 7^th^ 70–115, and apical segment 176–220 mm long, apical spine of antenna 11–20 mm long, subapical seta absent; fleshy sensory seta near apical seta 13–21 mm long; all segments of antenna covered with very robust, spine-like setae, the longest spine 21–37 mm long; first antennal segment with 0–2 spines on each side of segment. Eyes conical, well separated from base of first antennal segment.

*Venter*: Labium 285–350 mm long, apparently one segmented. Stylet loop usually longer than labium. Legs well developed; leg measurements: coxa length: front 186–310 mm long, middle 192–300 mm, hind 224–330 mm; front trochanter+femur 623–800 mm long, middle 647–820 mm, hind 705–883 mm; front tibia+tarsus 423–991 mm long, middle 448–994 mm, hind 548–1165 mm; front claw 82–106 mm long, middle 96–108 mm, hind 97–120 mm; hind claw digitules each 26–39 mm. Legs each with rows of robust spine-like setae, with a fleshy sensory seta on hind tibia, trochanter with 4 placoid sensilla on each surface. Wax plates present on ventral areas of head and thorax, and wide marginal plate laterad of each thoracic spiracle; two wax plates present between mid-coxae, both in triangular shape, anterior one relatively small and longer than wide (Fig. 6c); with 3 spine rows within ovisac band. Thoracic spiracles each with wide band of disc pores inside atrium. Setae few, scattered in medial areas of thorax, with several setae near anterior edge of ovisac band and associated with simple pores. Ovisac band narrow, indented in the middle. Multilocular pores each with 4 loculi around perimeter and 1 loculus in central hub, 4–5 mm in diameter; present in 3 complete bands near posterior edge of each spine band, and scattered around vulva.

*Dorsum*: Wax plates cover entire dorsum; three triangular wax plates on mid-dorsum, anterior 40–130 mm wide, 60‒195 mm long; middle 60‒120 mm wide, 90‒250 mm long; posterior 80‒110 mm wide, 90‒240 mm long, (Fig. 6d). Spines at margin of wax plate each 14–20 mm long, apically capitate. A few setae present in marginal clusters near posterior edges of marginal wax plates; with 3‒6 setae laterad of thoracic spiracles, longest seta 17–20 mm long; also present in very small numbers on other wax plates and in medial bare area. Multilocular pores generally each with 4 loculi around perimeter, one loculus in central hub, sparsely present in wax plates. Abdominal spiracles numbering 8 pairs on the margin, last 3 situated in posterior apical spine clusters. Anal ring 120–132 mm long and 118–146 wide, bearing 6 blunt anal ring setae, each seta 136 mm long.

#### Distribution.

Austria, Belgium, Canada, Czech Republic, Denmark (Faeroe Islands), Estonia, Finland, France, Georgia, Greece, Greenland, Iceland, Ireland, Italy, Japan, Mongolia, Norway, Poland, Romania, former Soviet Union, Spain, Sweden, Switzerland, U.K., Ukraine, U.S.A. (Alaska).

#### Ecology.

*Host plants*: *Alnus viridis* (Betulaceae), *Caltha* sp. (Ranunculaceae), *Capparis* sp. (Capparaceae), *Calamagrostis langsdorfii*, *Carex* sp., *Deschampsia caespitosa* (Poaceae), *Chrysanthemum alpinum*, *Hieracium* sp., *Hymogyne* sp., *Solidago* sp. (Asteraceae), *Dryas octopetala* (Rosaceae), *Gentiana* sp. (Gentianaceae), *Geranium* sp. (Geraniaceae), *Iris setose* (Iridaceae), *Racomitrium lanuginosum* (Grimmiaceae), *Calluna vulgaris*, *Rhododendron ferrugineum*, *Vaccinium myrtillus* (Ericaceae), *Saxifraga aizoon* (Saxifragaceae), *Saxifraga cuneifolia*, *Saxifraga oppositifolia*, *Sedum* sp. (Crassulaceae), *Soldanella* sp. (Primulaceae), *Trientalis europaea* (Myrsinaceae), and *Collybia* sp. (Fungi: Tricholomataceae). Under mosses (*Sphagnum* sp.: Sphagnaceae) and stones on roots, also reported from ant nests (Ben-Dov et al. 2014, Kozár 2004). In Primorsky region of Russia (Danzig 1980) and Japan, some individuals were observed on ferns.

### Arctorthezia occidentalis

Taxon classificationAnimaliaHemipteraOrtheziidae

(Douglas, 1891)

**Figure 3.**
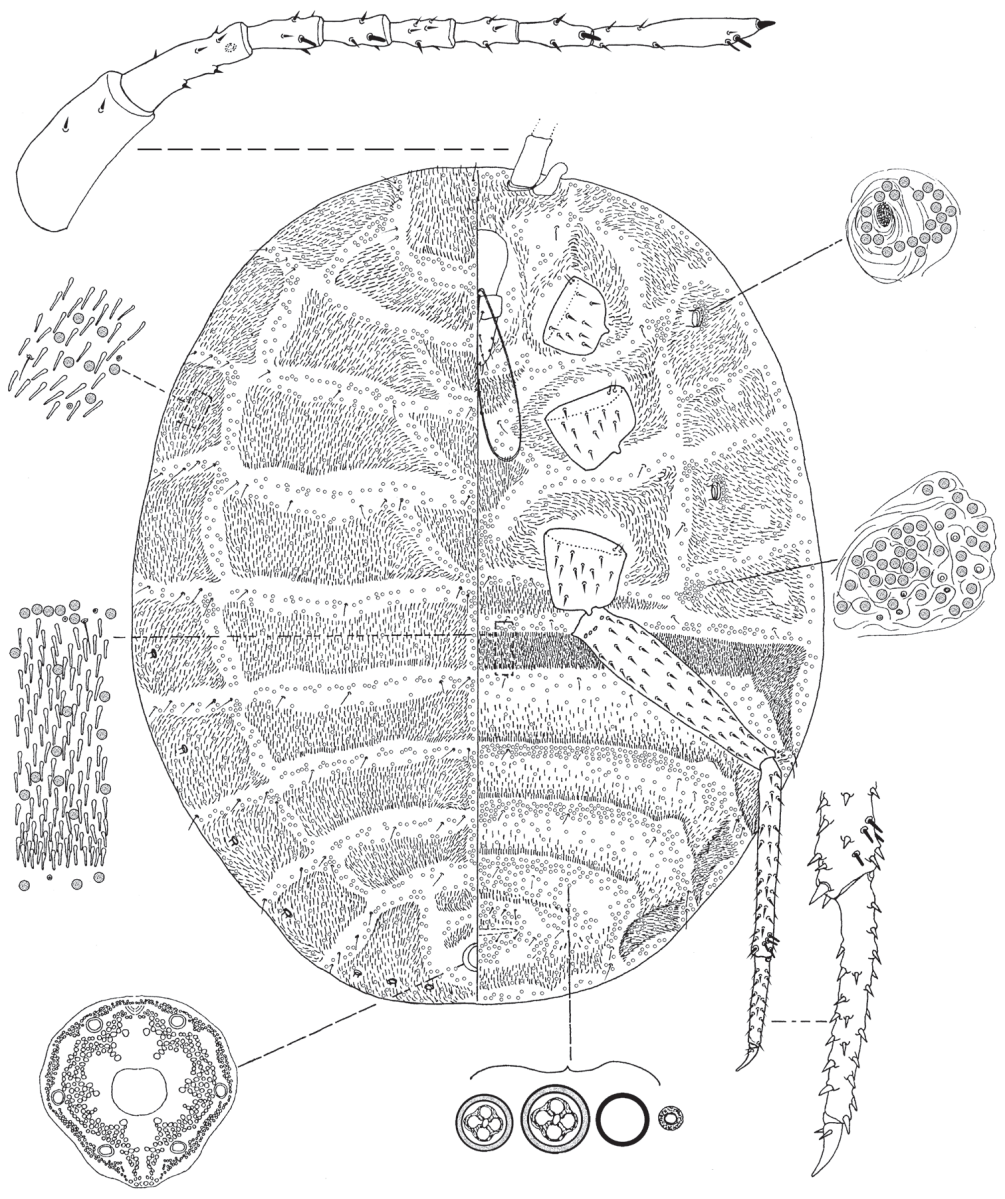
*Arctorthezia occidentalis* (Douglas, 1891), mounted adult female. Illustration by Konczné Benedicty & Kaydan.

Figs 3 , 5d–e , 6e–f

#### Material examined.

**Canada:** 2 females on 1 slide, British Columbia, Victoria, Highland Rd., moss, 12.vii.1988, leg. F. Kozár [PPI 3330, USNM 57]; 2 females on 1 slide, British Columbia, Vancouver Island, Ladysmith, entering a dwelling, 9.xi.1945, leg. W.A. Ross [PPI 8986, USNM 45-2340]; 6 females on 4 slides, British Columbia, Kaslo B.C., root of trees and grass, 19.v.1908, leg. J.W. Cockle [PPI 8974, USNM without code]; **USA:** 5 females on 5 slides, Montana, on apple,. ix.1949, leg. W.S. Regan [PPI 8975, USNM 49-2173]; 1 female, Colorado, Boulder, on *Castilleja* sp., 8.vi.1917, leg. H.F. Dietz [PPI 8977, USNM FHB #16096]; 4 females on 2 slides, Colorado, Boulder, on *Castilleja* sp., 12.vi.1919, leg. H.F. Dietz [PPI 8976, USNM without code].

#### Synonymy.

*Orthezia occidentalis* Douglas, *Orthezia californica* (Ehrhorn), Orthezia (Arctorthezia) occidentalis Morrison (Ben-Dov et al. 2014).

#### Description.

Adult female, with secretion, fairly large, 3.5 mm wide and 4.5 mm long. Body completely covered with dense, sharply defined wax plates, these occurring in the usual marginal and dorsal tufts, white or variously discoloured, sometimes appearing yellow-brown or grey (Morrison 1925). Live adult female with 9 paired dorsal wax plates in each marginal row, 8 in each submedian band, and 3 wide, triangular or shield-shaped plates in centre of thorax (Fig. 5d–e).

*Slide-mounted adult female* (Fig. 3, 6e–f). Body elongate oval, 3.45‒4.25 mm long, 3.00‒2.68 mm wide. Antenna 8- (rarely 7) segmented. Measurements of antennal segments: 1^st^ 280‒330, 2^nd^ 190‒230, 3^rd^ 110‒140, 4^th^ 100‒110, 5^th^ 90, 6^th^ 90‒100, 7^th^ 90‒120, and apical segment 235‒240 mm long; apical spine of antenna 20 mm long, subapical seta absent; fleshy sensory seta near apical seta 25 mm long, all segments of antennae covered with very robust, spine-like setae, longest spine 15 mm long, first antennal segment with 2 spines on each side of segment. Eye with sub-parallel sides, tall, 120‒180 mm long, situated very close to first segment of antenna.

*Venter*: Labium 340‒450 mm long, apparently one segmented. Stylet loop usually longer than labium. Legs well developed; measurements of front coxa 220‒260 mm long, middle 200‒250 mm, hind 240‒350 mm; front trochanter+femur 660‒720 mm long, middle 720‒820 mm, hind 760‒920 mm; front tibia+tarsus 460‒670 mm long, middle 540‒580 mm, hind 340‒400 mm; front claw 80 mm long, middle 90‒110 mm, hind 90‒110 mm; hind claw digitules 5‒10 mm; legs with rows of robust spine-like setae, with four fleshy sensory setae on tibia, trochanter with 4 placoid sensilla on each surface. Wax plates present on ventral areas of head and thorax, with wide marginal plate laterad of each thoracic spiracle; two wax plates present between mid-coxa upper one in triangular shape (Fig. 6e), below one knickers shape. Thoracic spiracles with wide band of disc pores inside atrium. Setae few, scattered in medial areas of thorax, with several setae near anterior edge of ovisac band and associated with simple pores. Ovisac band wide, straight in the middle; with 4 spine rows within ovisac band. Multilocular pores with 4 loculi around perimeter, 1 loculus in central hub; 5 mm in diameter; present in 3 complete bands near posterior edges of each spine bands, and scattered around vulva.

*Dorsum*: Wax plates cover all dorsum; three triangular wax plates on middorsum (first 340–540 mm in width, 140‒240 mm in length; middle 440‒740 mm in width, 300‒370 mm in length; third 640‒650 mm in width, 220‒330 mm in length, Fig. 6f). Spines at margin of wax plate 20‒22 mm long, spines apically capitate. A few setae present in marginal clusters near posterior edges of marginal wax plates, with 3‒6 setae laterad of thoracic spiracles, longest seta 40‒50 mm long also present in very small numbers on other wax plates and in medial bare area. Multilocular pores generally with 4 loculi around perimeter, one loculus in central hub, sparsely present in wax plates. Abdominal spiracles 7 pairs on the margin, last 2 in posterior apical spine clusters. Anal ring 130‒145 mm long and 100‒125 wide with 6 anal ring blunted setae, each 225‒250 mm long.

#### Distribution.

Canada (British Columbia), Spain, USA (Alaska, California, Colorado, Hawaiian Islands, Idaho, Montana, New Mexico, Oregon, Washington).

#### Ecology.

*Host plant*: *Bahia* sp., *Eriophyllum*, *Argyroxiphium* sp., *Eriophyllum confertifolium*, *Agrostis sandwicensis*, *Grossularia* sp., *Fragraria* sp. *Rubus* sp., *Castilleja* sp (Ben-Dov et al. 2014, Kozar 2004).

#### Biology.

On the roots, associated with *Formica integra*, and *Myrmica*? ants (Ben-Dov et al. 2014, Kozar 2004).

#### Comments.

Its presence in the Palaearctic Region (Spain) (Gómez-Menor 1937) is under question. Searches for slides in Spanish collections were unsuccessful (personal communication from Carolina Martin (Madrid), and Angeles Vásquez (Madrid)).

### Arctorthezia pseudoccidentalis

Taxon classificationAnimaliaHemipteraOrtheziidae

Morrison, 1952

**Figure 4.**
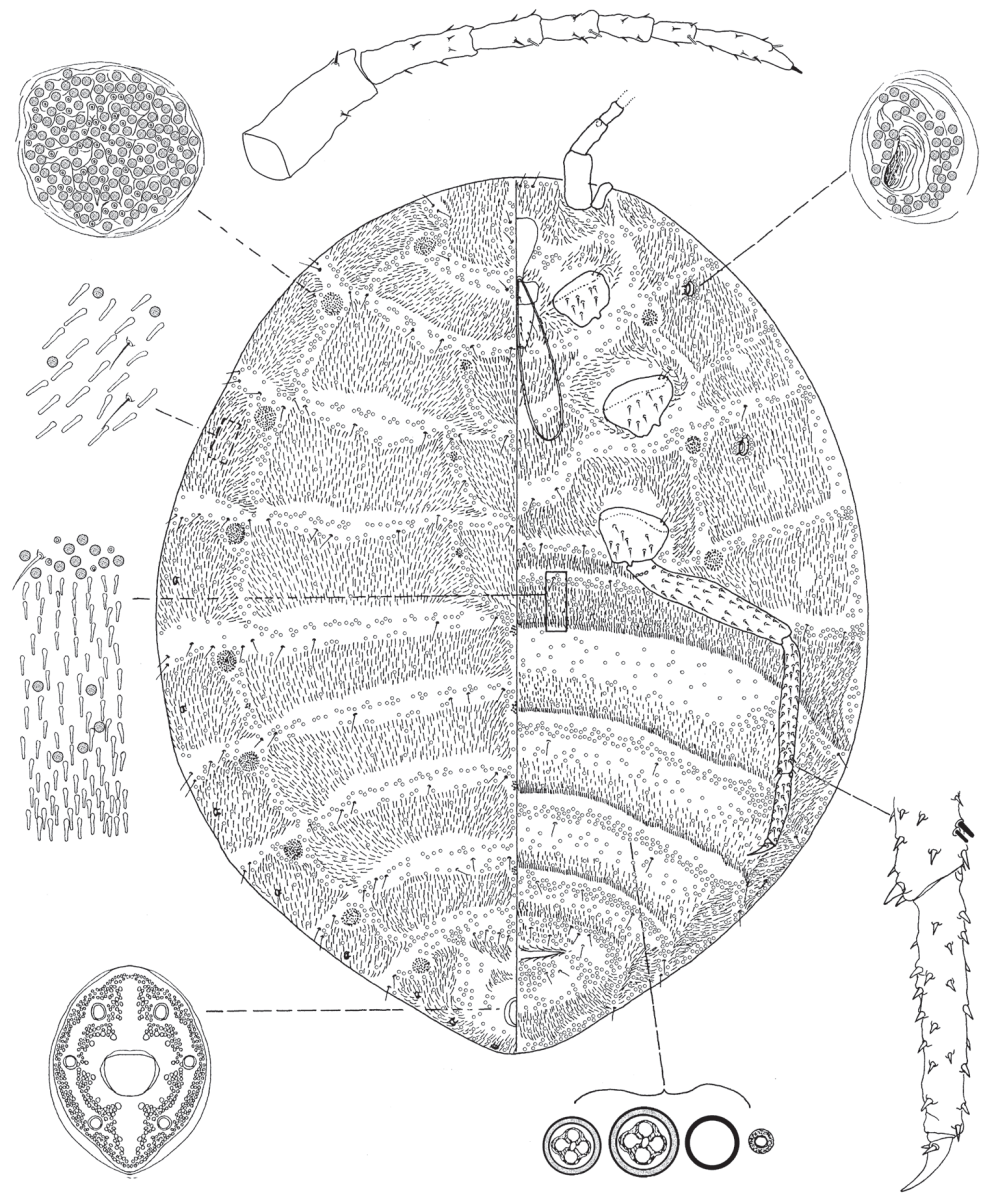
*Arctorthezia pseudoccidentalis* Morrison, 1952, mounted adult female. Illustration by Konczné Benedicty & Kaydan.

**Figure 5.**
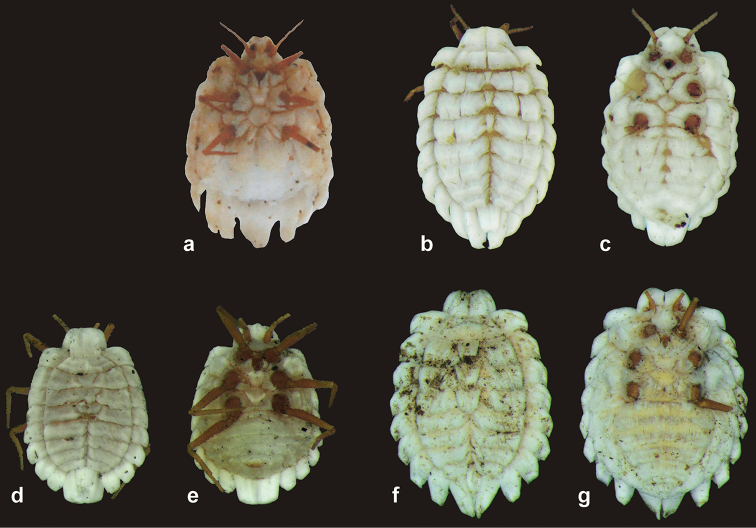
Dorsal and ventral views of unmounted adult female *Arctorthezia* species. *Arctorthezia helvetica* sp. n., **a** venter. *Arctorthezia cataphracta* **b** dorsum **c** venter. *Arctorthezia occidentalis* **d** dorsum **e** venter. *Arctorthezia pseudoccidentalis* **f** dorsum **g** venter. Photographs by: **a** P. van Helsdingen **b–g** É. Szita.

**Figure 6.**
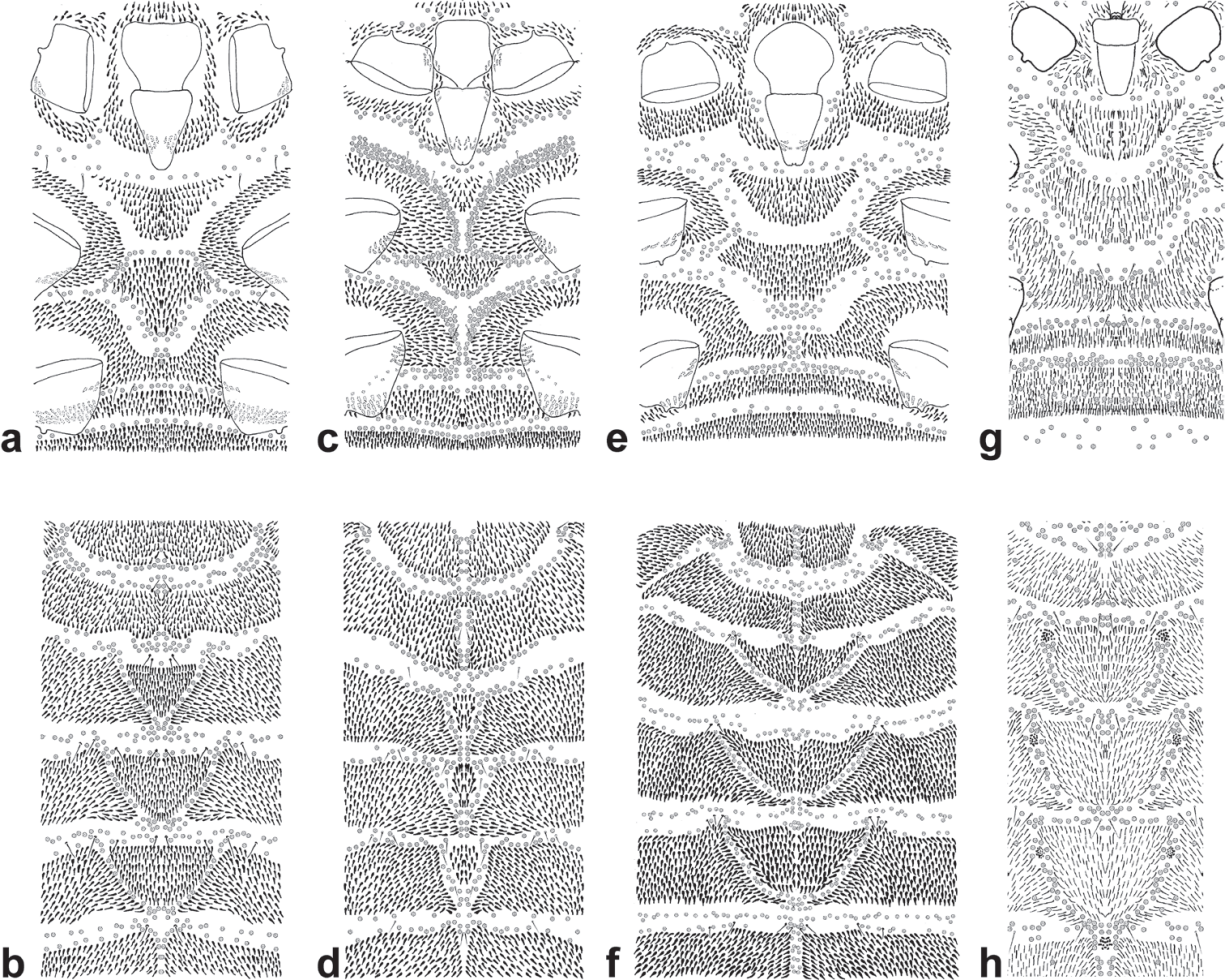
Enlargement of mid-dorsal and mid-ventral thoracic region of *Arctorthezia* species. *Arctorthezia helvetica* sp. n., **a** ventral **b** dorsal; *Arctorthezia cataphracta* **c** ventral **d** dorsal; *Arctorthezia occidentalis* **e** ventral **f** dorsal; *Arctorthezia pseudoccidentalis* **g** ventral **h** dorsal. Illustrations by Konczné Benedicty & Kaydan.

Figs 4 , 5f–g , 6g–h

#### Material examined.

**USA:** 4 females on 4 slides, Nevada, 15 mi S of Ely, beneath boards, 6.vii.1960, leg. T.R. Haig [PPI 8964, USNM 60-0381]

#### Description.

Adult female, with secretion, fairly large, similar to *Arctorthezia occidentalis*. Body completely covered with dense, sharply defined wax plates, these occurring in the usual marginal and dorsal tufts, white. With 3 wide, shield-shaped plates in centre of thorax (Fig. 5f–g).

*Slide-mounted adult female* (Fig. 4, 6g–h). Body elongate oval, 4.015 mm long, 3.497 mm wide. Antenna 7 or 8 segmented. Measurements of antennal segments: 1^st^ 320‒350, 2^nd^ 227‒260, 3^rd^ 144‒155, 4^th^ 113–124, 5^th^ 103, 6^th^ 93–103, 7^th^ 103, apical segment 191 mm long; apical spine of antenna 26 mm long, subapical seta absent; fleshy sensory seta near apical seta 18 mm long; all segments of antennae covered with very robust, spine-like setae, longest spine 11 mm long; first antennal segment with 2 spines on each side of segment. Eye with sub-parallel sides, tall, situated very close to first segment of antenna.

*Venter*: Labium 391‒402 mm long, apparently one segmented. Stylet loop usually longer than labium. Legs well developed; measurements of front coxa 247‒310 mm long, middle 309‒330 mm, hind 330‒340 mm; front trochanter+femur 917 mm long, middle 958 mm, hind 1060‒1088 mm; front tibia+tarsus not seen, middle 649 mm, hind 824–868 mm; front claw 1 not seen, middle 108 mm, hind 113 mm; hind claw digitules 23–26 mm; legs with rows of robust spine-like setae, without 2 fleshy sensory seta on tibia each 10 mm, trochanter with 4 placoid sensilla on each surface. Wax plates present on ventral areas of head and thorax, with wide marginal plate laterad of each thoracic spiracle; two wax plates present between mid-coxa in wide triangular shape, below one wide arch shape, with 4 spine rows within ovisac band (Fig. 6g). Thoracic spiracles with wide band of disc pores inside atrium. Setae few, scattered in medial areas of thorax, with several setae near anterior edge of ovisac band and associated with simple pores. Ovisac band wide. With four wide wax plate bands within ovisac band. Multilocular pores with 4loculi around perimeter, 1 loculus in central hub; 5 mm in diameter; present in 4 complete bands near posterior edges of each spine bands, and scattered around vulva.

*Dorsum*: Wax plates cover all dorsum; three shield-shaped setae plates on middorsum (first 390‒540 mm in width, 290‒400 mm in length; middle 540‒710 mm in width, 330‒460 mm in length; third 570‒720 mm in width, 370‒460 mm in length, Fig. 6h), 9 circular pores clusters, separating the dorsal and marginal wax plate bands. Spines at margin of wax plate 22‒27 mm long, spines apically capitate. A few setae present in marginal clusters near posterior edges of marginal wax plates, with 3‒6 setae laterad of thoracic spiracles, longest seta 40‒50 mm long also present in very small numbers on other wax plates and in medial bare area. Multilocular pores generally with 4 loculi around perimeter, one loculus in central hub, sparsely present in wax plates. Abdominal spiracles 8 pairs on the margin, last 3 in posterior apical spine clusters. Anal ring 161 mm long and 127‒138 wide with 6 anal ring blunted setae, each 259‒264 mm long.

#### Distribution.

USA (California, Idaho, Nevada, Washington).

#### Ecology.

*Host plant*: *Berberis aquifolium* (Berberidaceae). Grass roots, litter under different trees, often under rocks.

#### Concluding comment.

Macromorphological characters of ortheziid species, such as the shape, number and arrangement of the wax plates of live (or dead) ensign scales have been useful for genus and in some cases for species identification (Kondo et al. 2013, Kozár 2004, Szita et al. 2010). These wax plates on the intact body (Fig. 5) do not always have the same appearance as the wax plates of the mounted female (Figs 1–4, 6). For example, the submedial dorsal wax plates of *Arctorthezia cataphracta* are apparently in two parts on the intact body (Fig. 5b), while on the mounted female there is only one undivided wax plate on each tergite in the same position (Fig. 2). In the case of *Arctorthezia* species both the usability and limitations of this method for separating species within the genus are clear; the medial wax plates and midcoxal wax plates can be useful characters for quick separation of species (Figs 5–6). Nevertheless, the correct identification of a species needs always to be confirmed by slide mounting.

## Supplementary Material

XML Treatment for Arctorthezia

XML Treatment for Arctorthezia helvetica

XML Treatment for Arctorthezia cataphracta

XML Treatment for Arctorthezia occidentalis

XML Treatment for Arctorthezia pseudoccidentalis
